# The proteasome inhibitor MG132 reduces immobilization-induced skeletal muscle atrophy in mice

**DOI:** 10.1186/1471-2474-12-185

**Published:** 2011-08-15

**Authors:** Annabelle Z Caron, Sonia Haroun, Élisabeth Leblanc, Frédéric Trensz, Chantal Guindi, Aziz Amrani, Guillaume Grenier

**Affiliations:** 1 Centre de Recherche Clinique Étienne-Lebel, 3001-12th Avenue North, Sherbrooke, QC, J1H 5N4, Canada; 2 Department of Orthopedic Surgery, 3001-12th Avenue North, Sherbrooke, QC, J1H 5N4, Canada; 3 Department of Medicine, Université de Sherbrooke, 3001-12th Avenue North, Sherbrooke, QC, J1H 5N4, Canada

## Abstract

**Background:**

Skeletal muscle atrophy is a serious concern for the rehabilitation of patients afflicted by prolonged limb restriction. This debilitating condition is associated with a marked activation of NFκB activity. The ubiquitin-proteasome pathway degrades the NFκB inhibitor IκBα, enabling NFκB to translocate to the nucleus and bind to the target genes that promote muscle atrophy. Although several studies showed that proteasome inhibitors are efficient to reduce atrophy, no studies have demonstrated the ability of these inhibitors to preserve muscle function under catabolic condition.

**Methods:**

We recently developed a new hindlimb immobilization procedure that induces significant skeletal muscle atrophy and used it to show that an inflammatory process characterized by the up-regulation of TNFα, a known activator of the canonical NFκB pathway, is associated with the atrophy. Here, we used this model to investigate the effect of in vivo proteasome inhibition on the muscle integrity by histological approach. TNFα, IL-1, IL-6, MuRF-1 and Atrogin/MAFbx mRNA level were determined by qPCR. Also, a functional measurement of locomotors activity was performed to determine if the treatment can shorten the rehabilitation period following immobilization.

**Results:**

In the present study, we showed that the proteasome inhibitor MG132 significantly inhibited IκBα degradation thus preventing NFκB activation in vitro. MG132 preserved muscle and myofiber cross-sectional area by downregulating the muscle-specific ubiquitin ligases atrogin-1/MAFbx and MuRF-1 mRNA in vivo. This effect resulted in a diminished rehabilitation period.

**Conclusion:**

These finding demonstrate that proteasome inhibitors show potential for the development of pharmacological therapies to prevent muscle atrophy and thus favor muscle rehabilitation.

## Background

Skeletal muscle atrophy may be caused by prolonged immobilization, which has a significant impact on the duration and intensity of rehabilitation [1]. It is characterized by increased muscle fatigability associated with changes in muscle fiber size, reduced muscle protein synthesis [2], and enhanced muscle protein ubiquitination and degradation [3,4]. The pro-inflammatory cytokine TNFα has been reported to be involved in the process of muscle wasting by causing myofibrillar protein degradation via the ubiquitin proteasome pathway [5]. By activating transcription factor NFκB, TNFα plays a central role in regulating muscle protein catabolism in vitro and in vivo [6-8]. Mourkioti et al. have shown that muscle genetically depleted of NFκB exhibits increased strength, diminished protein degradation under atrophic conditions, and enhanced regeneration in response to injury [9]. Moreover, it has recently been reported that muscle atrophy caused by NFκB activation is associated with the induction of MuRF-1 [8,10] and Atrogin/MAFbx [11], two markers of atrophy [12,13]. Interestingly, we recently showed that immobilization induced-atrophy is characterized by an up-regulation of the pro-inflammatory cytokines TNFα, IL-1, and IL-6 concomitantly with an up-regulation of MuRF-1 and Atrogin/MAFbx [14]. The idea that inflammation may play a significant role in inducing skeletal muscle atrophy has also been proposed by Hirose et al. [15] and Andrianjafiniony et al. [16].

Inactive NFκB is retained in the cytoplasm through its association with IκBα. However, when stimulated with pro-inflammatory cytokines (TNFα, IL-1, IL-6), IκBα is phosphorylated, ubiquitinated, and degraded through the proteasome machinery, allowing free NFκB to translocate to the nucleus to transactivate its target genes [17]. The inhibition of the proteasome machinery prevents the degradation of IκBα, which maintains NFκB in its inactive state [18], thus preventing the up-regulation of MuRF-1 and Atrogin/MAFbx. The present study suggested that the NFκB canonical pathway plays a central role in immobilization-induced skeletal muscle atrophy and that proteasome inhibitors prevent muscle atrophy by maintaining NFκB in an inactive state.

Interestingly, proteasome machinery inhibitors reduce skeletal muscle proteolysis in vitro [19-21]. Proteasome inhibitors (Velcade™ and MG132) also prevented muscle mass loss in an in vivo rat model of skeletal muscle wasting induced by denervation and cast immobilization of the hindlimb [22,23]. However, it is unclear whether the prevention of muscle mass loss by proteasome inhibitors can lead to functional.

To explore the effect of proteasome inhibitors on preventing muscle atrophy following hindlimb immobilization, we investigated the mechanism by which the proteasome inhibitor MG132 affects the NFκB canonical pathway in the C2C12 myogenic cell line. We also explored the effect of MG132 in an in vivo mouse model of skeletal muscle wasting induced by immobilization in which the tibialis anterior (TA) muscle undergoes rapid atrophy. Time to exhaustion experiments showed that proteasome inhibition markedly accelerates the rate of rehabilitation following immobilization. In summary, the effect of MG132 indicated that proteasome inhibitors show promise as pharmacological agents for preventing muscle atrophy and for favoring the rehabilitation of patients following severe trauma or surgery.

## Methods

### Animals and muscle atrophy-induced procedure

Sixteen-week-old male CD1 mice (Charles River, Montreal, QC, Canada) were used for all our experiments. Thirty minutes before the immobilization procedure, 0.1 mg/kg of buprenorphine was administrated IP. The mice were then anesthetized using isoflurane. The right hindlimb was immobilized as previously described [14]. Briefly, the hindlimb was immobilized 7 days by stapling the foot exploiting normal dorso-tibial flexion using an Autosuture Royal 35W skin stapler (Tyco Healthcare, Pointe Claire, QC, Canada). One tine was inserted close to the toe at the plantar portion of the foot while the other was inserted in the distal portion of the gastrocnemius. The other hindlimb was used as a control. During the immobilization period, the mice were injected subcutaneously with MG132 (7.5 mg/kg/dose) or vehicle (DMSO) twice daily. DMSO containing or not MG132 was diluted in sterile pure corn oil (1:100, injected volume 150 μL). After 7 days, the tibialis anterior (TA) muscles of immobilized and non-immobilized hindlimbs from MG132- and DMSO-treated mice were either harvested or unstapled for remobilization studies. MG132 or DMSO was given only during the immobilization period.

Cast immobilization was performed according to an adapted procedure described by Frimel et al. [24]. Briefly, hindlimbs were immobilized in the neutral position (160-180°). The ankle was fixed at 90° to keep the TA resting. The plaster of Paris cast encompassed both hindlimbs as in a hip spica. A thin layer of padding was placed underneath the cast in order to prevent abrasions. With the ankles at a straight angle, the cast did not have to be tight to prevent the mice from getting out of the cast. Care was taken to minimize the weight of the cast.

All animal experiments were approved by the Animal Ethics Committee of Université de Sherbrooke and were performed in accordance with Canadian Council on Animal Care guidelines (protocol #133-06B).

### Cell culture and treatment

C2C12 cells were cultured in complete proliferation medium consisting of DMEM supplemented with 10% heat-inactivated fetal bovine serum (FBS), 100 IU/ml of penicillin, and 100 μg/ml of streptomycin. Differentiation was initiated by replacing the cell growth medium with differentiation medium (DMEM supplemented with 5% horse serum and antibiotics). After 96 h, the C2C12 cells had differentiated into myotubes and were treated with 40 μM MG132 in DMSO or vehicle (DMSO). After a 1-h treatment, the cells were stimulated or not with TNFα (50 ng/ml) in presence or absence of MG132.

### Measurement of NFκB transcriptional activity

C2C12 cells were cultured in six-well tissue culture plates (3 × 10^5 ^cells/well) and transiently co-transfected with an NFκB-luciferase reporter plasmid (pNFκB-luc, 1 μg, a generous gift from Dr. T. E Gunter, University of Rochester, NY, USA) and a control *Renilla *luciferase plasmid (pRL-CMV, 30 ng, Promega, Madison WI, USA) in Lipofectamine 2000 transfection reagent (Invitrogen, Burlington, ON, Canada). Twenty-four hours after the transfection, the growth medium was replaced by differentiation medium, and the incubation was continued for an additional 96 h. Differentiated cells were then treated with MG132 (40 μM) or vehicle (DMSO). After a 1-h treatment, the cells were stimulated or not with TNFα (50 ng/ml). Six hours later, the cells were washed twice with PBS and lysed to determine transcriptional activity using a reporter assay system (Promega, Madison, WI, USA) and a SIRIUS luminometer (Berthold Detection Systems, Pforzhein, Germany).

#### Electrophoresis and immunoblotting

Protein extracts from C2C12 myotubes were solubilized in RIPA buffer (0.5% NP-40, 0.1% sodium deoxycholate, 150 mM NaCl, 50 mM Tris-HCl, pH 7.5) supplemented with Complete™ Protease Inhibitor Cocktail (Roche Molecular Biochemicals, Laval, QC, Canada). The homogenates were centrifuged at 14,000 rpm for 10 min at 4°C and the supernatants were collected. The protein concentrations in the supernatants were measured using Bradford's method (BioRad). The protein extracts (50 μg) were separated on a 12% polyacrylamide gel and electrotransferred to a polyvinylidene fluoride membrane (PVDF) (Millipore, Bedford, MA, USA). Blotted membranes were incubated overnight at 4°C in PBS-T (8 mM NaH_2_PO_4_, 1.5 mM KH_2_PO_4_, 3.5 mM KCl, 137 mM NaCl) containing 0.1% Tween-20 with anti-IκBα (1:1,500) or anti-GAPDH antibody (1:1,000) (Santa Cruz Biotechnology, Santa-Cruz, CA, USA). After extensive washing with PBS-T, the blots were incubated for 1 h at room temperature with a peroxidase-conjugated secondary antibody. After extensive washing with PBS-T, the immunostained bands were revealed with ECL Plus according to the manufacturer's instructions on a BioMax ML film (Kodak, Rochester, NY, USA). The autoradiograms were digitized and the bands were quantified by densitometric measurements using ImageJ software [25].

### Quantitative PCR

Total RNA was extracted from flash-frozen crushed TA muscle and C2C12 myotubes using TRIzol^® ^reagent according to the manufacturer's instructions. The RNA was reverse-transcribed using Reverse Transcriptase Superscript II. qPCR assays were performed using 50 ng of template cDNA. The conditions for all the reactions were as follows: an initial 5 min denaturation step at 95°C, followed by forty 40-s cycles at 95°C, 56°C, and 72°C. The qPCR assays were performed using a Rotor-Gene 6000 (Corbett Robotics, Australia) and iQSYBR Green Supermix (BioRad). Results were calculated using the 2^-ΔΔCT ^relative quantification method normalized to the HPRT1 gene. The primer sets are shown in Table 1.

**Table 1:** Primer sets used for quantitative PCR analyses

Gene	NCBI Accession Number	Forward Primer	Reverse Primer	Product Size (bp)
**Hprt1**	NM_013556.2	5'- gcaaactttgctttccctgg -3'	5'- acttcgagaggtccttttcacc -3'	85

**MuRF-1**	DQ229108	5'-tgcctggagatgtttaccaagc-3'	5'-aaacgacctccagacatggaca-3'	143

**Atrogin-1/MAFbx**	AF441120	5'-aaggctgttggagctgatagca-3'	5'-cacccacatgttaatgttgccc-3'	223

**Il-1β**	NM_008361	5'-gcccatcctctgtgactcat-3'	5'-aggccacaggtattttgtcg-3'	230

**Il-6**	NM_031168	5'-agttgccttcttgggactga-3'	5'-tccacgatttcccagagaac-3'	159

**TNFα**	NM_013693	5'-ccgatgggttgtaccttgtc-3'	5'-tggaagactcctcccaggta-3'	217

qPCR conditions for all reactions included an initial 5 min denaturation step at 95°C, followed by forty 40-s cycles at 95°C, 56°C, and 72°C.

### Histology

TA muscles were excised, fixed in formalin, and embedded in paraffin. Sections (5 μm) were stained with hematoxylin and eosin (H&E). The H&E-stained sections were used for the cross-sectional area (CSA) analyses. Over 40 myofibers/field from at least six different fields were examined (20× magnification) using an optical microscope (TE-2000-S; Nikon, Mississauga, ON, Canada) and the myofiber cross-sectional areas were measured using ImageJ software [25].

### Functional measurement of locomotor ability - Running time

The locomotor ability (running time) of mice that had both hindlimbs immobilized for 7 days before the staples were removed was measured using a treadmill (Panlab/Harvard Apparatus, Barcelona, Spain). The mice were divided into groups of 9-10 animals each. The groups received either twice-daily subcutaneous injections of MG132 in canola oil (7.5 mg/kg/dose) or vehicle (DMSO in canola oil) immediately after the immobilization procedure and then for the duration of the 7-day immobilization period. We compared the two groups to a group of non-hindlimb-immobilized age-matched mice. The running time experiment was performed using a two-lane motorized treadmill equipped with shocker plates. The treadmill was run at an inclination of +25° at 8 m/min for 5 min, after which the speed was increased to 10 m/min for 2 min and then 20 m/min. The test was stopped when the mouse remained on the shocker plate for more than 15 s without attempting to reengage the treadmill. The running time was determined from the beginning of the test. The running time experiments were performed 4 and 11 days after removing the staples and on days 0 and 7 for the non-hindlimb-immobilized age-matched control mice.

### Statistics

All data are expressed as means ± SEM. Paired *t-*tests were used to assess the statistical significance of differences between treated muscles and untreated contralateral muscles. Unpaired *t-*tests were used to compare two groups of mice or two different time points. A *p *< 0.05 was considered statistically significant. Statistical values were calculated using GraphPad Prism 5.0 software™.

## Results

### MG132 inhibited TNFα-induced proteasome activation in differentiated C2C12 cells

In a previous study, we showed that immobilization-induced skeletal muscle atrophy is associated with the upregulation of the inflammation markers TNFα, IL-6, and IL-1 [14]. The stimulation of TNFα, a well-known activator of the NFκB canonical pathway, causes the rapid degradation of IκBα through proteasome activation, resulting in the release of NFκB, which translocates to the nucleus where it binds to the target genes that promote muscle atrophy [26].

**Figure 1:**
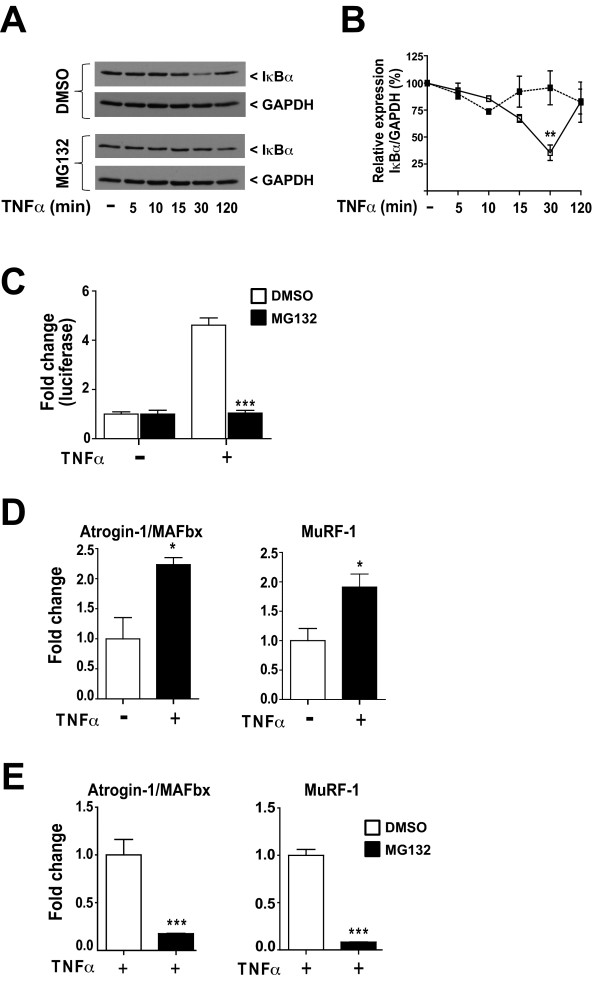
**MG132 inhibited TNFα-induced proteasome activation in differentiated C2C12 cells**. **(A) **Western blot showing IκBα expression in differentiated C2C12 pre-treated with vehicle (DMSO) or MG132 for 1 h followed by stimulation with TNFα (50 ng/ml) for 5, 10, 15, 30, and 120 min in presence or absence of MG132. GAPDH was used as a loading control. **(B) **Graphical representation of IκBα expression levels over time indicating that a pre-treatment with MG132 (dashed line) was able to prevent IκBα degradation compared to cells pre-treated with the vehicle (DMSO) (solid line). **(C) **C2C12 myoblasts co-transfected with the NFκB-luciferase and *Renilla *luciferase reporter vectors. They were then differentiated and stimulated with TNFα for 6 h in the presence or absence of MG132. The ratio of Luciferase:*Renilla *activities indicates that the presence of MG132 prevented the binding of NFκB to the luciferase promoter. **(D) **Graphs showing MuRF-1 and Atrogin/MAFbx qPCR expression data from C2C12 myotubes stimulated or not with TNFα (50 ng/ml) for 1 h. TNFα caused the expression of the muscle-specific ubiquitin ligases MuRF-1 and Atrogin/MAFBx. **(E) **To evaluate the impact of MG132 on MuRF-1 and Atrogin/MAFbx expression, differentiated C2C12 were pre-treated or not with MG132 (40 μM) for 1 h before being stimulated with TNFα (50 ng/ml) for 1 h. Expression of MuRF-1 and atrogin/MAFbx was quantified by qPCR. MG132 inhibited the expression of MuRF-1 and atrogin/MAFbx. Data are presented as the means ± SEM of at least four independent experiments performed in triplicate. (*p < 0.05, **p < 0.005, ***p < 0.001 compared to control).

Since the inhibition of the proteasome can prevent IκBα degradation and NFκB translocation to the nucleus of many cell types [27,28], we first investigated whether this was the case for myotubes from the differentiated mouse myogenic cell line C2C12. We measured IκBα protein levels by Western blot analysis in terminally differentiated C2C12 cells exposed to TNFα in the absence or presence of the proteasome inhibitor MG132. In the presence of TNFα, the amount of IκBα was significantly lower after 30 min in terminally differentiated C2C12 cells (35 ± 7%) compared to TNFα-treated cells exposed to MG132 (Figure 1). After 120 minutes, the amount of IκBα protein was greater than after 30 min and had almost returned the initial basal level (Figures 1A and 1B). A densitometry analysis clearly indicated that IκBα was rapidly degraded after 30 min but rebounded after 120 min of TNFα stimulation (Figure 1B). These results can be consistent with the previously reported de novo synthesis of IκBα[18].

We used an NFκB-dependent luciferase reporter assay to confirm that MG132 suppressed NFκB activity by inhibiting the proteasomal degradation of IκBα. C2C12 myoblasts transfected with the NFκB-luciferase reporter gene were terminally differentiated for 5 days before TNFα (50 ng/ml) was added in the absence or presence of MG132 (40 μM). Six hours of stimulation with TNFα caused a 4.6 ± 0.3-fold induction of pNFκB-luciferase activity in control cells compared to cells treated with MG132 (Figure 1C), indicating that the inhibition of the proteasome by MG132 was sufficient to inhibit the degradation of IκBα and thus reduce the transcriptional activity of NFκB in terminally differentiated C2C12 cells.

The exposure of muscle to an inflammatory milieu is known to accelerate protein degradation that may occur via the ubiquitin proteasome pathway [29-32]. We thus determined whether TNFα can induce atrophy in terminally differentiated C2C12 cells by measuring the expression of the muscle-specific ubiquitin ligases atrogin-1/MAFbx-1 and MuRF-1 genes, two molecular markers of atrophy. There was a significant induction of MuRF-1 (1.9 ± 0.2 fold) and atrogin-1/MAFbx-1 mRNA (2.2 ± 0.1 fold) in cells stimulated with TNFα for 1 h compared to unstimulated cells (Figure 1D). To investigate the impact of MG132 on the expression of MuRF-1 and atrogin-1/MAFbx-1 mRNA, cells were treated with MG132 for 1 h prior to being stimulated for 1 h with TNFα. MG132 dramatically decreased the induction of both MuRF-1 (0.08 ± 0.001) and atrogin-1/MAFbx-1 (0.16 ± 0.006) in the presence of TNFα (Figure 1E), suggesting that TNFα promotes atrophy via a proteasome-dependent pathway.

Our results also revealed that the stimulation of TNFα led to the activation of the NFκB canonical pathway in the differentiated myogenic cell line C2C12. This inflammatory stimulus can induce the up-regulation of both MuRF-1 and Atrogin/MAFbx, which are responsible for muscle protein loss during atrophy. Interestingly, MG132 inactivated NFκB and, concomitantly, the expression of MuRF-1 and atrogin-1/MAFbx-1.

### MG132 inhibited the key pathways involved in muscle atrophy

**Figure 2:**
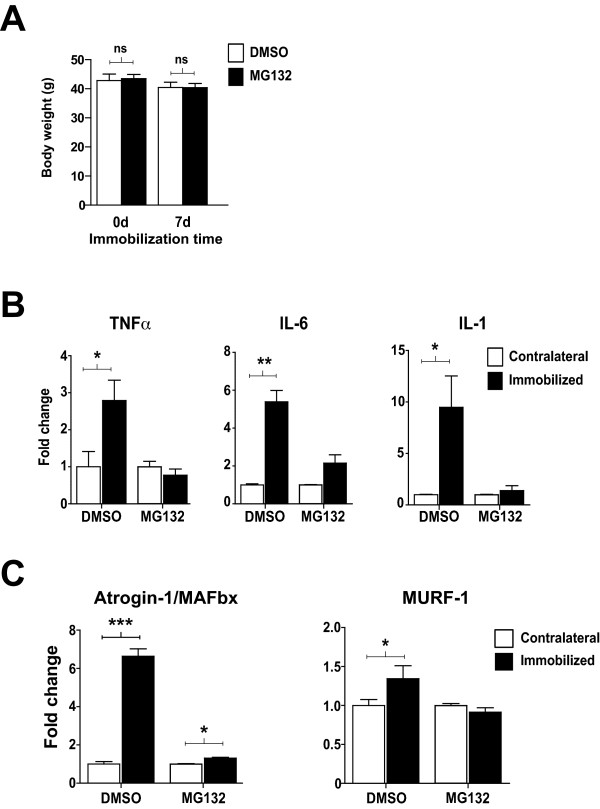
**MG132 inhibited the key pathways involved in muscle atrophy**. **(A) **Histogram showing the body weights of MG132- and DMSO-treated mice at day 0 (n = 8) and day 7 (n = 8) of immobilization using staples. As can be seen, there was no difference in total mean body weight between the MG132- and DMSO-treated mice. **(B) **TNFα, IL-6, and IL-1 mRNA levels were measured by qPCR. Data show a robust expression of TNFα, IL-6, and IL-1 in the immobilized hindlimb compared to the non-immobilized contralateral hindlimb of the DMSO-treated mice. Immobilization-induced mRNA expression was totally inhibited in the MG132-treated mice (n = 8 mice/condition). **(C) **qPCR expression analyses showing that MG132 was able to prevent the up-regulation of MuRF-1 and Atrogin/MAFbx in immobilized hindlimbs compared to DMSO-treated mice (n = 8 mice/condition). Data are presented as means ± SEM. (*p < 0.05, **p < 0.005, ***p < 0.001 compared to control).

In a previous study, we described an original immobilization procedure for inducing skeletal muscle atrophy. The atrophy was associated with the presence of inflammation and the up-regulation of muscle-specific ubiquitin ligases [14]. Since MG132 was able to down-regulate the expression of MuRF-1 and atrogin-1/MAFbx-1 in terminally differentiated C2C12 cells, we next evaluated the in vivo effect of MG132 on the expression of these ligases in our immobilization-induced skeletal muscle atrophy model. TA muscles from mice in which one hindlimb was immobilized for 7 days while the contralateral was not. The mice were injected subcutaneously with MG132 or DMSO (vehicle) for 7 days of immobilization and the TA muscles were then harvested. There was no significant difference between the total mean body weights of the MG132- and DMSO-treated mice after 7 days of immobilization using staples (Figure 2A). qPCR analyses revealed a robust and significant increase in the expression of TNFα, IL-6, and IL-1 (2.79 ± 0.55, 5.39 ± 0.59, and 9.48 ± 3.05 fold, respectively) in the immobilized TA muscle compared to contralateral TA muscle of vehicle-treated mice (Figure 2B). Interestingly, the atrophy caused by a plaster cast (see additional file 1A-B), also revealed a significant increase in the same inflammatory factors within the TA muscles 24 h, 3.5 d, and 7 d post-immobilization (see additional file 1C), which was concomitant with the expression of the muscle-specific ubiquitin ligases (see additional file 1D). It should be noted that the expression of the inflammatory molecules was not due to the presence of the staple or the dorso-tibial flexion position of the foot, which suggests that inflammation observed in the staple model was due to the immobilization. However, there were no significant differences in TNFα, IL-6, and IL-1 mRNA levels in the immobilized and non-immobilized contralateral TA muscles from the MG132-treated mice, indicating that MG132 was able to inhibit the expression of inflammation markers known to be regulated by the NFκB canonical pathway.

The ability of our hindlimb immobilization model to induce skeletal muscle atrophy is related to the increase in MuRF-1 and atrogin-1/MAFbx-1 levels (1.34 ± 0.17- and 6.64 ± 0.39-fold, respectively) (Figure 2C). No significant difference was observed between MuRF-1 mRNA levels in immobilized and non-immobilized contralateral TA muscles from MG132-treated mice. However, the MG132 treatment significantly reduced the mRNA expression of atrogin-1/MAFbx-1 as compared to the control (DMSO-treated) immobilized TA muscles. Together these results suggest that MG132 can prevent the onset of the inflammatory process and can also contribute to the down-regulation of MuRF-1 and atrogin-1/MAFbx-1, two critical modulators of muscle atrophy.

### Immobilization-induced atrophy was prevented by MG132

**Figure 3:**
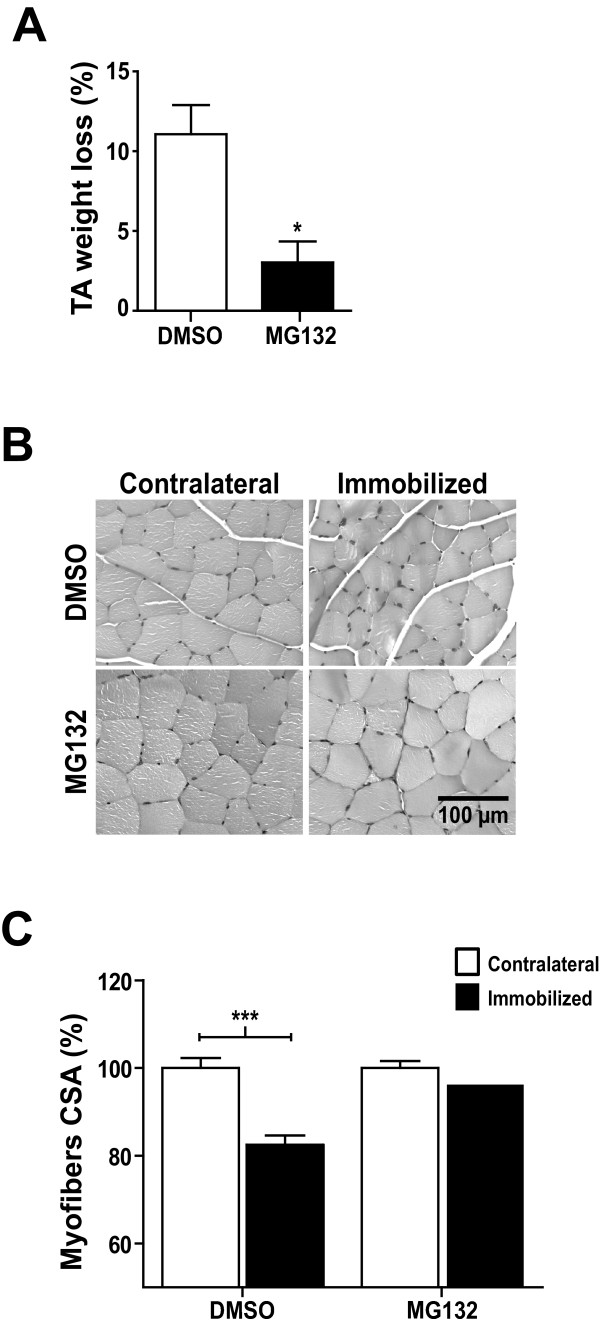
**Immobilization-induced atrophy was prevented by MG132**. **(A) **Graph showing the percentage of atrophy of tibialis anterior (TA) muscles from the hindlimbs of MG132- and vehicle (DMSO)-treated mice after 7 days of immobilization. The percentage of TA atrophy was calculated by comparing each immobilized TA muscle to the respective non-immobilized contralateral TA muscle such that each animal served as its own control (n = 16). MG132 significantly attenuated the loss of muscle mass due to immobilization at day 7 post-immobilization. **(B) **H&E-stained cross-sections of immobilized and non-immobilized contralateral TA muscles from MG132- and vehicle (DMSO)-treated mice. The results show that MG132 prevented myofiber atrophy in the TA muscles. **(C) **Histogram showing the percentage area of myofiber (CSA) of TA muscles from immobilized and non-immobilized contralateral TA muscles isolated from MG132- (non-immobilized contralateral, n = 536 myofibers; immobilized, n = 643 myofibers) and DMSO-treated mice (non-immobilized contralateral, n = 241 myofibers; immobilized n = 302 myofibers). MG132 maintained the fiber size of immobilized TA muscles. Data are presented as means ± SEM. For the CSA of muscle myofibers, data are expressed as means ± SEM. (*p < 0.05, **p < 0.005, ***p < 0.001 compared to control)

Skeletal muscle atrophy is characterized by a reduction in skeletal muscle mass and myofiber diameter, two features that are readily evident following hindlimb immobilization [14]. Since MG132 significantly reduced muscle ubiquitin ligase levels, thus causing myofibrillar degradation, we investigated the effect of MG132 on the TA muscles of immobilized and non-immobilized contralateral hindlimbs. After 7 days of immobilization, we observed an increase in the percentage of weight loss (11.1 ± 1.8%) calculated from the contralateral TA muscles in DMSO-treated mice (Figure 3A). Interestingly, the MG132 treatment significantly reduced the percentage of weight loss (atrophy) in TA muscles from immobilized hindlimbs, with a loss of muscle wet mass of only 3.04 ± 1.3% in MG132-treated mice, or 3.6-fold less than in DMSO-treated mice (Figure 3A).

To better characterize the effect of MG132 on muscle mass wasting, we performed histological analyses of TA muscles. H&E staining of TA muscle paraffin cross-sections revealed that immobilization induced significant atrophy in DMSO-treated mice as shown by the considerable reduction in myofiber CSA. As expected, the myofiber CSA of TA muscles from MG132-treated mice appeared to be the same for both immobilized hindlimbs and non-immobilized contralateral hindlimbs (Figure 3B). Image analyses showed that the CSA of myofibers of TA muscles from DMSO-treated mice had decreased to 82.5 ± 2.1% compared to 96.2 ± 2.1% to the CSA of myofibers of TA muscles from MG132-treated mice (Figure 3C). These measurements were obtained by comparing the CSA of immobilized TA muscles to CSA of non-immobilized contralateral TA muscles, which was arbitrary fixed at 100%. In addition, histological analyses revealed that there were no significant compound-related adverse effects in skeletal muscle from MG132-treated mice (Figure 3B).

These results indicate that MG132 was able to prevent immobilization-induced skeletal muscle atrophy as shown by the preservation of muscle mass and the maintenance of myofiber size.

### MG132 diminished the recovery time of muscle following hindlimb immobilization

**Figure 4:**
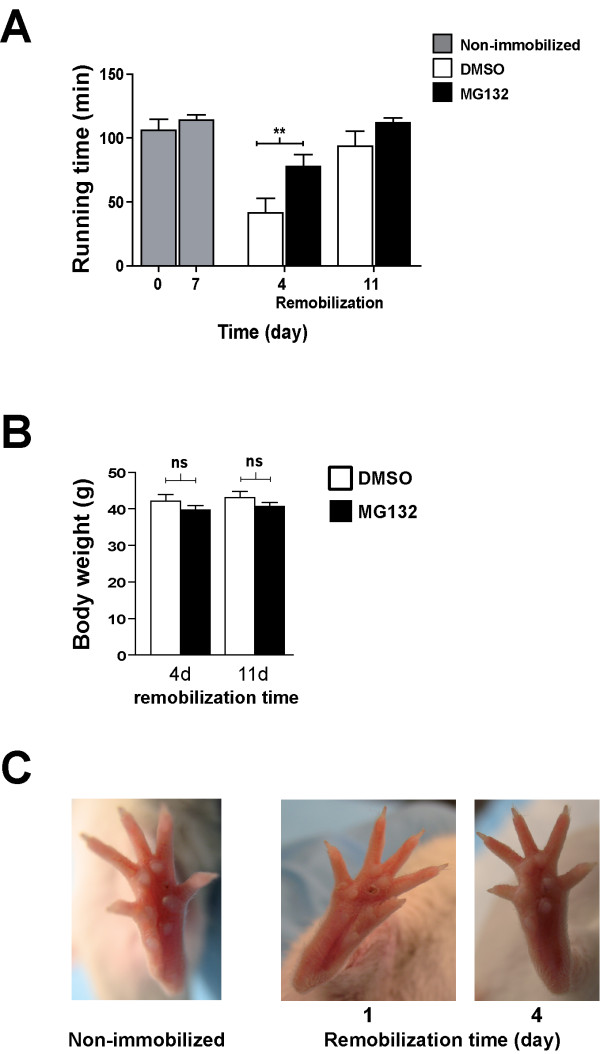
**MG132 diminished the recovery time of muscle following hindlimb immobilization**. **(A) **The mice were immobilized using surgical staples (both hindlimbs) with or without the MG132 treatment. After 7 days, the staples were removed and the mice were remobilized. Running time experiments were performed using the treadmill. Histogram showing that the MG132-treated mice (n = 10) displayed a longer physical performance on day 4 post-remobilization compared to the DMSO-treated mice (n = 10). However, there was no significant difference on day 11 post-remobilization (n = 9-10 mice/condition). In addition, there was no significant difference, after a 7 day remobilization period, with the physical performance of age-matched control mice (gray column) that had never been immobilized, indicating that training did not improve the physical performance (n = 5). Data are presented as means ± SEM. (*p < 0.05, **p < 0.005, ***p < 0.001 compared to control). **(B) **Histogram showing the body weights of the same MG132- and DMSO-treated mice after 4 (n = 8) and 11 (n = 8) days of remobilization. As can be seen, there was no difference in total mean body weight between the MG132- and DMSO-treated mice. **(C) **Photographs showing the ventral part of the foot in which the staple had been inserted. A slight insult was observed 1 day after the staple was removed (1 day of remobilization). After 4 days of remobilization, which corresponded to a time point in the treadmill performance experiment, the insult had completely disappeared.

In a previous study, we showed that running performance was lower following hindlimb immobilization and that this could be reversed during the remobilization period [14]. Since MG132 prevented skeletal muscle atrophy following hindlimb immobilization, we investigated whether MG132 had an impact on muscle strength and locomotor ability per se. We performed a running time experiment with mice that had both hindlimbs immobilized. The mice were separated into two groups. The first group was treated with vehicle (DMSO) and the second, with MG132. After 7 days of immobilization, the staples were removed, and the hindlimbs were remobilized after which functional recovery was assessed (Figure 4A). After 4 days of remobilization, the DMSO-treated mice had a significantly shorter running time (27.6 ± 14.8 min) than the MG132-treated mice (76.6 ± 13.4 min) (Figure 4A). After 11 days of remobilization, there was no significant difference in the running time of DMSO- and MG132-treated mice (83.8 ± 18.2 min and 115.4 ± 3 min, respectively) (Figure 4A). Interestingly, there was no significant difference in the running time of the MG132-treated mice after 4 and 11 days of remobilization (76.6 ± 13.4 versus 115.4 ± 3 min), whereas it took 11 days for the DMSO-treated mice to completely recover, suggesting that MG132 reduced the effect of immobilization-induced atrophy, which was reflected by the longer running time of MG132-treated mice than of DMSO-treated mice after 4 days of remobilization. The body weights remained stable for 11 days following remobilization (Figure 4B).

To verify whether the running time values of the mice following remobilization were optimal, we added a control group of mice that had never had their hindlimbs immobilized. The running time of the control group was not significantly different from that of mice that had been remobilized for 11 days, indicating that the mice had completely recovered from the 7-day immobilization after 11 days of remobilization.

We also showed that the surgical staple immobilization procedure had little impact on the ventral side of the foot. A slight insult was observed 1 day after staple was removed (1 day of remobilization) and had completely disappeared after 4 days of remobilization, indicating that the immobilization procedure likely did not change the capacity of the mice to perform the running time experiment on the treadmill (Figure 4C).

## Discussion

Skeletal muscle atrophy caused by immobilization is a major challenge for patient rehabilitation. The development of therapies designed to prevent or attenuate skeletal muscle atrophy will fill an important medical need. Many therapeutic trials focusing on the TNFα-NFκB pathway have been undertaken with the purpose of reducing the impacts of muscle diseases. For example, anti-TNFα antibodies (Remicade™) have been show to interfere with TNFα activity and consequently reduce the breakdown of dystrophic muscles [33]. A systemic treatment with curcumin, an NFκB inhibitor, has been shown to stimulate muscle regeneration after traumatic injury [34]. The use of a synthetic double-stranded oligodeoxynucleotide as a cis-element to block the binding of NFκB to promoter regions has been shown to inhibit cachexia in a mouse tumor model [35]. Another strategy to interfere with the NFκB signaling pathway involves the use of proteasome inhibitors. Proteasome inhibitors have been shown to inhibit IκBα degradation and prevent NFκB activation [27,28]. Since the activity of the NFκB canonical pathway relies on the proteasome machinery, we sought to determine whether the use of the proteasome inhibitor MG132 during immobilization-induced stimulus would enhance physical performance.

We recently showed that the inflammatory molecule TNFα is involved in skeletal muscle atrophy using an immobilized hindlimb mouse model [14]. TNFα is known to increase the activity of the canonical NFκB pathway in conditions associated with muscle weakness, including sepsis, cancer, and ageing [36-39] and to cause protein degradation in cultured myotubes [5,6]. In fact, the ubiquitin-proteasome pathway has been shown to be of major importance in the catabolic signaling of TNFα leading to the breakdown of muscle protein [40]. Ubiquitinated proteins are selectively targeted for degradation through the 26S proteasome pathway by tissue-specific E3 ligases [12,13] that catalyze the transfer of activated ubiquitin. The muscle-specific E3 ligases MuRF-1 and Atrogin/MAFbx are part of atrophy [13]. A review of the literature revealed slight differences in the results regarding the genes involved in the proteasome degradation pathway when it is perturbed by an increase in NFκB activity. MuRF1 mRNA, but not Atrogin/MAFbx mRNA, is upregulated in the gastrocnemius muscle of transgenic mice with constitutive NFκB activity [8]. Judge et al. electrotransferred a dominant negative IκBα into the soleus muscle of mice that were subsequently tested in an unloading atrophy model. They showed that Atrogin/MAFbx but not MuRF-1 is regulated by the NFκB pathway in the soleus muscle [11]. On the other hand, Bodine et al. (2001) used disuse, denervation, and hindlimb suspension models of atrophy to show that both MuRF1 and Atrogin/MAFbx are upregulated in the gastrocnemius muscle [13]. These slight differences may be due to the different types of muscle studied, the backgrounds of the animals used, and the different models used to induce muscle atrophy.

In the present work, we showed that the proteasome inhibitor MG132 interfered with the NFκB canonical pathway of TNFα-treated C2C12 cells by protecting IκBα from degradation and thus preventing the activation of NFκB transcription. Lastly, we showed that MG132 prevents the TNFα-induced up-regulation of MuRF1 and Atrogin-1/MAFbx in vitro. Our results demonstrated that MG132 protects IκBα from degradation, which confirmed the central role of the proteasome machinery in the activation of the NFκB canonical pathway [41,42].

We used an immobilization model that induced skeletal muscle atrophy to show that a treatment with the proteasome inhibitor MG132 prevented the immobilization-induced atrophy of the TA muscle. These results correlated with the histological analysis showing that MG132 preserved myofiber size during immobilization. The proteasome inhibitor Velcade™ (also known as PS-341 or Bortezomib™) has been shown to partially but significantly reduce the denervation-induced atrophy of the soleus muscle [22]. The authors concluded that the partial inhibition was due to an inadequate dose of Velcade™ or other proteolytic mechanisms that are not inhibited by the drug, such as the Ca^2+^-dependent protease or lysosomal pathways, which are also known to be involved in muscle wasting [43]. In addition to inhibiting proteasome activity, MG132 has been reported to repress certain lysosomal cysteine proteases and calpains [44], which might also explain the total rescue of muscle mass in our study.

The immobilization-induced model is known to cause an early inflammatory process characterized by the up-regulation of TNFα, IL-1, and IL-6 mRNA, which are targets of the canonical NFκB pathway [45-47]. Interestingly, treating mice with MG132 prevented the up-regulation of these pro-inflammatory genes during immobilization. Likewise, the levels of MuRF-1 and Atrogin/MAFbx mRNA, which are targets of NFκB, increased after immobilization. This effect was prevented by MG132, which completely inhibited the expression of MuRF-1 mRNA. Surprisingly, unlike the in vitro experiment, MG132 did not completely inhibit Atrogin/MAFbx mRNA expression in immobilized mice, but did significantly attenuate it. We cannot rule out the involvement of another mechanism in the increase in Atrogin/MAFbx mRNA levels in vivo. There is some evidence suggesting that disuse muscle atrophy induced by unloading is associated with the activation of an alternative NFκB pathway distinct from the pathway seen with cachexia where TNFα is responsible for muscle atrophy [48,49]. Despite the fact that we observed an up-regulation of the canonical NFκB pathway target genes for TNFα, IL-1, and IL-6 [45-47], it is possible that there is a cross-over between with the non-canonical NFκB pathway, which is independent of IκBα. Other investigators have shown that TNFα mediates the upregulation of atrogin/MAFbx expression in the skeletal muscle via p38MAPK [10,50]. These conflicting findings suggest that multiple signaling pathways mediate muscle wasting, and highlight the complexity of the molecular pathways involved in the regulation of skeletal muscle atrophy.

To correlate the morphological, histological, and molecular results with the functional properties of TA muscles from MG132-treated mice, we evaluated the time to exhaustion performance of mice. Our results showed that the endurance of MG132-treated mice was significantly higher than that of vehicle-treated control mice after 4 days of remobilization. However, no significant differences were observed after 11 days of remobilization. Indeed, after 11 days, the time to exhaustion was similar to that of mice that had never had their hindlimbs immobilized.

## Conclusion

In summary, we showed that the proteasome inhibitor MG132 is able to inhibit the activation the NFκB pathway by inhibiting the degradation of IκBα in vitro. We showed that MG132 prevent the up-regulation of TNFα, IL-1 and IL-6 mRNA, which are target genes of the canonical NFkB pathway in the TA muscle of hindlimb immobilized mice. A direct consequence of the MG132 treatment was the repression of MuRF-1 and Atrogin/MAFbx, which may explain why the loss of muscles mass and the decrease in myofiber CSA was lower in the TA muscles of MG132-treated mice. To our knowledge, this is the first study to clearly show that preventing atrophy by inhibiting the proteasome can shorten the rehabilitation period following immobilization. In conclusion, we showed that proteasome inhibitors protect muscle integrity and functionality. The use of pharmacological agents directed against the proteasome may be an avenue of great interest for developing therapies to prevent muscle atrophy and improve the rehabilitation of patients following severe trauma or surgery.

## List of abbreviations

CSA: cross sectional area; DMSO: dimethyl suloxide; TA: tibialis anterior; Tnfα: tumor necrosis factor alpha.

## Competing interests

The authors declare that they have no competing interests.

## Authors' contributions

AZC and GG designed the study and drafted the manuscript. AZC, SH, FT, EL, CG performed the experimental work and the statistical analysis. AA participated in study design. All authors have read and approved the final manuscript.

## Pre-publication history

The pre-publication history for this paper can be accessed here:

http://www.biomedcentral.com/1471-2474/12/185/prepub

## Supplementary Material

Additionnal file 1 **Cast immobilization induced inflammation and the expression of muscle-specific ubiquitin ligase**. **(A) **Photograph showing a mouse hindlimb immobilized using a plaster cast. The hindlimb is in the neutral position. **(B) **Histogram showing the percentage of weight loss of TA after 7 days of immobilization as compared to TA from non-immobilized hindlimbs (n = 4). **(C) **Graphs showing RNA expression levels of the inflammatory markers TNFα, IL-6, and IL-1. The levels of these markers were significantly higher at 24 h, 3.5 d, and 7d post-cast immobilization than in non-immobilized hindlimbs. **(C) **Graphs showing the expression of MuRF1 and Atrogin-1 transcripts. The levels of MuRF-1 and Atrogin-1 transcripts were significantly higher at 24 h, 3.5 d, and 7 d post-immobilization than in non-immobilized hindlimbs. n = 4/time studied (*p < 0.05, **p < 0.005, ***p < 0.001 compared to control). Click here for file
